# Moyamoya Disease in Pregnancy: Management after Intracranial Bypass Grafting

**DOI:** 10.1155/2012/638471

**Published:** 2012-03-26

**Authors:** A. C. Gimovsky, C. J. Macri, S. L. Bathgate, D. E. Ross

**Affiliations:** ^1^Department of Obstetrics and Gynecology, The George Washington University Medical Center, 2150 Pennsylvania Avenue, NW, Washington, DC 20037, USA; ^2^Department of Neurology, University of Mississippi Medical School, Jackson, MS 39216, USA

## Abstract

Moyamoya disease (MD) is a chronic, progressive cerebrovascular disease distinguished by bilateral stenosis or occlusion of the arteries around the circle of Willis with resulting prominent arterial collateral circulation. We describe a pregnant woman in whom this diagnosis was confirmed by cerebral angiogram and treated with bilateral superficial temporal artery-middle cerebral artery (STA-MCA) bypass grafting prior to conception. The patient was managed with strict blood pressure monitoring and low-dose aspirin antepartum, intrapartum, and postpartum. The patient presented in spontaneous labor at term and underwent a spontaneous vaginal delivery without complications.

## 1. Introduction

 MD is a progressive cerebrovascular disease distinguished by bilateral stenosis or occlusion of the arteries supplying the circle of Willis and resulting in a prominent arterial collateral circulation. The disease is characterized by intimal thickening in the walls of the internal carotid arteries bilaterally. Intraventricular, subarachnoid, and intracerebral hemorrhage has been described with MD. There is a paucity of the literature regarding management during pregnancy. We present a case of a woman with MD who underwent intracranial bypass grafting prior to conception and subsequently underwent a successful vaginal delivery. 

## 2. Case

 A 30-year-old right-handed African American presented for prenatal care at 10 weeks of gestation. The patient had a past medical history that was significant for a “stroke” in 1999. In 2008, the patient presented to the emergency department (ED) with difficulty using her right hand and transient left-sided weakness and numbness. A preliminary diagnosis of transient ischemic attack (TIA) was made. Her evaluation at that time included an MRI which demonstrated encephalomalacia in the right frontal cortex; MRA at that time demonstrated “irregular vessels” but was otherwise interpreted as unremarkable.

**Figure 1:**
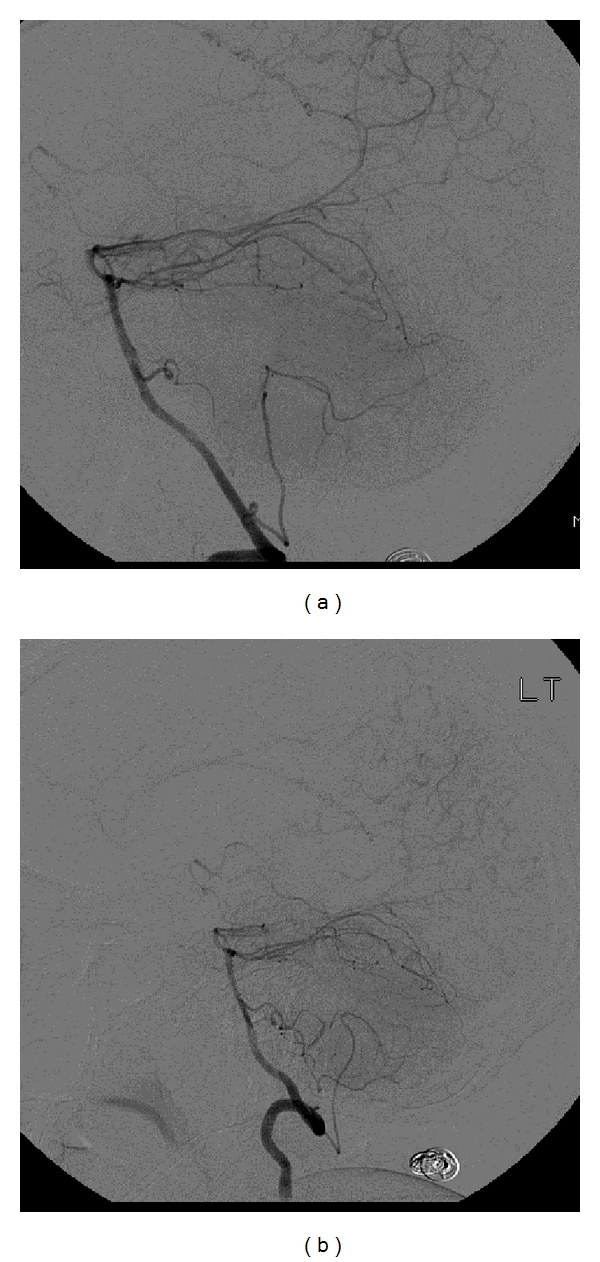
The attached pictures are digitally subtracted images from a cerebral angiogram of our patient. (a) Right vertebral injection showing irregular, serpiginous ill-defined vessels. (b) Left vertebral injection showing similar findings.

A follow-up evaluation by a neurosurgeon confirmed the diagnosis of MD based upon findings at a cerebral angiogram. Please see Figures 1(a) and 1(b) for images from her cerebral angiogram. The patient underwent bilateral STA-MCA bypass grafting. One month following the second procedure the patient presented to the ED with aphasia and right hemiplegia. Evaluation revealed a left basal ganglia hemorrhage. Her symptoms subsequently resolved. 

The patient was asymptomatic upon her presentation to George Washington University Medical Center Prenatal Clinic at 10 weeks of pregnancy. Her blood pressure was 136/86 and her weight 263 pounds. The patient had stopped taking low-dose aspirin when she learned she was pregnant, but low-dose aspirin was subsequently re-initiated. Her pregnancy was managed with home blood pressure monitoring, and her BP remained less than 140/90 throughout gestation. No antihypertensive medications were necessary. Ultrasound demonstrated a normal fetus, and serial ultrasounds documented normal growth. 

The patient presented in spontaneous labor at term. An epidural anesthetic was placed, and she underwent an unremarkable labor. Her second stage lasted 1 hour, and she was not actively managed. Blood pressure was monitored every 30 min and remained at less than 140/90. She had a spontaneous vaginal delivery without complications of a 2850 gram female infant with Apgar scores of 9 and 9 at one and five minutes, respectively.

## 3. Discussion

MD is a rare chronic progressive cerebrovascular disease distinguished by bilateral stenosis or occlusion of the arterial supply to the circle of Willis. The etiology of MD is unknown, but there is a higher incidence among the Japanese and other Asian populations. A familial inheritance occurs in approximately 10 percent of cases [1]. These cases are theorized to be inherited as an autosomal dominant with incomplete penetrance [2]. A specific gene locus has been described on chromosome 17 although involvement of chromosomes 3, 6, 8, and 12 has been reported by others [2].

“Moyamoya” is a Japanese word meaning puffy or hazy, like “a puff of smoke in the air.” The term was originally used to describe the angiographic appearance of the affected vascular collateral network [3]. In epidemiologic surveys conducted in Japan, the prevalence of Moyamoya has been found to be 3.2 to 10.5 per 100,000 population, with a slight female predominance [4–6]. In an American study, the breakdown of prevalence based on ethnicity as ratio to whites was 4.6 for Asian Americans, 2.2 for African Americans, and 0.5 for Hispanics [7].

Clinical features of MD include cerebrovascular events such as TIA, infarction, and hemorrhagic stroke. Diagnosis is suggested by cranial CT or MRI. Diagnosis utilizing vascular imaging with MRA, CTA, or conventional cerebral angiography is confirmed by demonstrating the stenotic or occlusive lesions found in the distal internal carotid arteries and the arteries proximal to the circle of Willis. Pathologically, MD appears as concentric stenosis or occlusion in the distal internal carotid arteries and large vessels of the circle of Willis. The vessels appear as a meshwork of overgrown and dilated small arteries.

There is no definitive treatment for Moyamoya disease other than neurosurgical palliation. Individuals experiencing TIAs and strokes are prescribed aspirin, vasodilators, or anticoagulants to reduce the risks of future recurrent neurological sequelae [8].

There have been at least 57 cases of MD in pregnancy described in the literature. 34 patients had been diagnosed with MD prior to pregnancy and 23 patients were symptomatic and diagnosed initially as having MD associated with pregnancy [9, 10]. The majority of cases reported have initially presented as an initial intracranial hemorrhage with neurological dysfunction [11, 12].

Autoregulation of cerebral blood flow is challenged during pregnancy by hypervolemia, hyperventilation, and a wide range of systemic blood pressures particularly in the third trimester. The presence of a vascular disease like MD poses a potential risk of ischemia and/or cerebral hemorrhage, during pregnancy, labor, or delivery.

Few reports of the management of pregnancy and delivery in pregnancy associated with MD are available for review. Authors agree that blood pressure must be strictly controlled [13].

Medical management includes antiplatelet treatment and blood pressure control. As in our patient, aspirin therapy was continued. Theoretically, aspirin can decrease risk of microemboli, which is a possible cause of ischemic disease in Moyamoya [14]. Risk of thrombosis is also increased in pregnancy; thus, aspirin may even be more essential during this period. Calcium channel blockers and magnesium sulphate may be best choices to control hypertension in Moyamoya because of their vasodilatory effects, which can reduce gravity of ischemic episodes [14]. Additionally, in pregnancy, calcium channel blockers and magnesium may have the extra benefit of treating pregnancy-related hypertension or preventing seizures in preeclampsia.

The preferred mode of delivery remains unclear. Cesarean delivery was chosen in the majority of previously reported cases. This approach has been suggested to better control fluctuations in blood pressure and potential alterations in cerebral blood flow that may occur during the active and second stage of labor. Elective cesarean delivery has been suggested to be the delivery mode of choice of the initial symptoms and diagnosis of MD was made during pregnancy [15]. Since cerebral blood flow is minimally affected by neural influences and the resultant effects of anesthesia may not play as large of a role in changes of intracranial pressure as they do in systemic pressure, not all authors are in agreement [16].

Given the dramatic increase in intravascular volume that accompanies placental separation regardless of the mode of delivery, vaginal delivery with regional anesthesia would seem to be a reasonable approach. Women with MD having had vaginal deliveries without neurological sequelae have been reported [10, 17]. Regional anesthesia is generally regarded as the preferred modality of analgesia as it provides easier monitoring for neurological changes as well as decreasing the risk of significant hypertension associated with intubation required for general anesthesia.

The present patient had two prior neurosurgical procedures. There is limited experience reported with intracranial vascular grafting under any circumstances in pregnancy. Patients who have undergone revascularization procedures were still at a significant risk for bleeding with MD [12].
